# Student-Run Online Journal Club Initiative During a Time of Crisis: Survey Study

**DOI:** 10.2196/33612

**Published:** 2022-03-07

**Authors:** Burak Berksu Ozkara, Mert Karabacak, Duygu Demet Alpaydin

**Affiliations:** 1 Cerrahpaşa Faculty of Medicine Istanbul University-Cerrahpaşa Istanbul Turkey

**Keywords:** online journal club, medical student, distance learning, COVID-19, undergraduate education, student journal club, online education, establishment, initiative, literature, research, publishing, education

## Abstract

**Background:**

Since the closure of university campuses due to COVID-19 in spring 2020 necessitated a quick transition to online courses, medical students were isolated from hospitals and universities, negatively impacting their education. During this time, medical students had no opportunity to participate in academic discussions and were also socially isolated. Furthermore, medical doctors and professors of medical schools were given additional responsibilities during the pandemic because they were the frontliners in the fight against COVID-19. As a result, they did not have enough time to contribute effectively to medical student education.

**Objective:**

This paper describes the establishment of the Cerrahpasa Neuroscience Society Journal Clubs, a group of entirely student-run online journal clubs at Cerrahpasa Faculty of Medicine, Istanbul University-Cerrahpasa.

**Methods:**

The website, mass emailing, and social media accounts were used to announce the online journal clubs. Only medical students were eligible to apply. Journal clubs included psychiatry, neuroradiology, neurosurgery, neurology, and neuroscience. Following the last journal club meeting, a questionnaire created by the society’s board was distributed to the participants. SPSS Statistics (version 26) was used for statistical analysis.

**Results:**

Since March 15, 2021, synchronous online journal club meetings have been held every 2 weeks on a weekday using Google Meet, Microsoft Teams, or Zoom. Meetings of each journal club lasted approximately 1 hour on average. Interstudent interaction across multiple institutions was achieved since a total of 45 students from 11 different universities attended the meetings on a regular basis. Students on the society’s board served as academic mentors for the clubs. The clubs received excellent feedback from participants, with an overall contentment score of 4.32 out of 5.

**Conclusions:**

By establishing these clubs, we have created a venue for academic discussions, which helps to reduce the negative impact of the pandemic on education. In addition, we believe it greatly aided students in staying in touch with their peers, thereby reducing the sense of isolation. We realize that traditional journal clubs are run by faculty; however, we believe that this experience demonstrated that medical students could run a journal club on their own since the feedback from participants was excellent. Additionally, as a medical student, being a journal club academic mentor is a challenging responsibility; however, having this responsibility significantly improved our academic mentors’ leadership abilities.

## Introduction

SARS-CoV-2, which is responsible for COVID-19, was declared a pandemic by the World Health Organization on March 11, 2020, posing new challenges for all medical students and faculty, as well as requiring remote learning by universities worldwide [1]. Platforms such as Zoom, Google Meet, and Microsoft Teams became the “new normal,” and the primary venue of teaching and socializing, in only a few weeks. This also influenced how journal clubs are run.

Since their introduction by Sir William Osler in 1875, journal clubs have a long history in the medical sciences [2]. Traditionally, they involved regular gatherings with a group of doctors and/or students to discuss publications. The original purpose was to help physicians stay up to date with current research and implement the research findings to clinical practice [3,4]. The concept of journal clubs evolved and widened as the depth of the literature continued to increase. As evidence-based learning has become more integrated in medical education, journal clubs have become a venue for not only keeping physicians up to date but also for dissecting and assessing the quality of study methodology [5].

Most journal clubs were still held in person prior to COVID-19. One significant problem of in-person journal clubs is that participants find it difficult to attend meetings on a regular basis, perhaps due to logistic challenges. This problem can be solved by using online platforms that allow participants to join the meeting from any location, thereby providing a flexible and feasible platform for evidence-based learning [6]. These meetings (ie, online journal clubs) can be held synchronously via platforms such as Google Meet or Microsoft Teams, or asynchronously via internet forums such as Twitter. Asynchronous virtual journal clubs can take place regardless of time or place; participants can contribute at a specified time period without waiting for the other participants to be online, which usually occurs on an internet blog. Online journal clubs, whether synchronous or asynchronous, enable national and international collaboration by bridging geographical boundaries [7]. They may also involve experts and even the authors of the discussed articles. In this way, online journal clubs create a venue that motivates medical professionals, increases networking, and minimizes social isolation, especially during the COVID-19 pandemic. It is worth noting that although the COVID-19 pandemic resulted in a rapid shift to online learning, online journal clubs have existed for a long time [8].

According to Keet et al [7], journal clubs are especially effective in resident training and continuing medical education. In addition, journal clubs can be very beneficial to medical students, especially when they are quarantined and forced to stay away from clinical environments during the pandemic. During this time of crisis, we believe that an online journal club could help medical students stay motivated, socialize, boost their medical knowledge, and teach them academic medicine. Since professors, lecturers, and other medical doctors were assigned additional responsibilities during the pandemic to aid in the battle against COVID-19, they were unavailable to run a journal club. Consequently, as the Cerrahpasa Neuroscience Society board, we have decided to establish and run a group of student-led synchronous online journal clubs. The aim of this article is to discuss the organization and outcomes of our online journal club experience based on survey results.

## Methods

### Ethics Considerations

This study was conducted in compliance with the principles of the Declaration of Helsinki. Ethical approval was waived because the Cerrahpasa Neuroscience Society’s journal clubs are run independently of the university. Informed consent was obtained from all participants.

### Clubs and Application

Cerrahpasa Neuroscience Society is a student-led organization that was founded in 2018 at Cerrahpasa Faculty of Medicine, Istanbul University-Cerrahpasa (IUC). In our society, we established five distinct online journal clubs with regard to the subfields of neuroscience: psychiatry, neuroradiology, neurosurgery, neurology, and neuroscience journal clubs. On February 19, 2021, all of the online journal clubs were announced to all Cerrahpasa Neuroscience Society email newsletter subscribers, as well as via postings on the Cerrahpasa Neuroscience Society’s Facebook, Twitter, and Instagram accounts. Between February 22 and 28, 2021, applications were accepted online at the society’s website for each journal club. Only pregraduate medical students were eligible to apply. Although the presentation language was announced to be Turkish, all of the reviewed papers were published in English. Therefore, applicants were reminded that English proficiency would be necessary for efficacious meetings. The number of participants in each journal club was intended to be between 9 and 12. The absolute number of participants was decided after the applications were submitted. Students on the society’s board served as academic mentors for the clubs. The board members of the society include Atacan Zeybek, Batuhan Davuş, Elif Kaymaz, Ferit Ulaş Özkan, Kardelen İnan, Naz Bilaloğlu, Öykü Melek Tepe, Zeynep Sude Furkan, Zeynep Özcan, Burak Berksu Özkara, Mert Karabacak, and Duygu Demet Alpaydın. The board members were selected via voting by the members of the Cerrahpasa Neuroscience Society based on their performance and contribution to the club in the 2019-2020 education year.

### Mission

Careful planning and the establishment of clear, defined goals are essential for a successful journal club [7]. Therefore, before the sessions began, the following goals were declared to the participants: to create an ideal discussion environment, to improve critical appraisal skills, to learn to review the literature, to learn to select appropriate articles, and to improve presentation skills. In an attempt to reach our goals, the society’s board also provided mentors with educational materials on how to run a journal club.

### Articles

Initially, each academic mentor selected articles for their participants to present based on their interests in a particular topic relevant to the journal club they run. All selected articles were in English, published in a well-respected journal, and possibly appealing to the students. However, after providing educational material on how to perform a literature review, a few academic mentors chose to provide presenters with autonomy by allowing them to choose what to present, which is an idea to motivate participants based on self-determination theory (SDT). SDT explains motivational processes and inspired us to foster this element of learning [9]. Furthermore, academic mentors presented one article in their first meetings to serve as an example and to share key components of an ideal presentation.

### Questionnaire and Analysis: Evaluation of Cerrahpasa Neuroscience Society Journal Clubs

Two authors (MK and DA) created a questionnaire that was controlled and evaluated by the first author (BO) on July 3, 2021. The questionnaire was sent via email to participants who attended more than 80% of the meetings, with a response deadline of July 9, 2021. To receive their certificate of participation, participants were required to complete the questionnaire, which resulted in a 100% response rate. The questionnaire was divided into three sections. The first section asked participants about their universities, current grades, journal clubs they attended, and the platform they used during meetings. The second section included 22 items that questioned participants’ level of contentment with our journal club. The third section included 8 items pertaining to the preferences of the participants (Table 1). Questions in sections 2 and 3 were assessed on a 5-point Likert scale, with 1 representing “completely disagree” and 5 representing “completely agree.” Negatively worded questions were reverse-scored (1=5, 2=4, etc).

SPSS Statistics (version 26) was used for statistical analysis. Because the first section of the questionnaire was about the demographics and the third section considered the preferences of the attendees, all statistics were calculated based on the second section of the questionnaire. The internal consistency of the questionnaire’s second section was assessed using Cronbach α. Independent-sample *t* tests were used to investigate group differences. Pearson correlations were used to investigate bivariate relationships between items of the questionnaire. *P* values less than .05 were regarded as statistically significant.

**Table 1:** Questionnaire items for evaluation of Cerrahpasa Neuroscience Society journal clubs.

Question number		Question
**Contentment questions**		
	Q1	Objectives of the journal club (provision of ideal discussion medium, development of critical-thinking skills, choice of up-to-date articles, efficient presentation) were explained clearly prior to meetings.
	Q2	Objectives of the journal club were achieved.
	Q3	Peer education in journal club meetings was more favorable in comparison to classical medical education.
	Q4	I would like to see journal clubs in other medical schools.
	Q5	Interval between meetings was sufficient for preparing presentations.
	Q6	Interval between meetings was sufficient for reading three papers.
	Q7	Articles chosen were compatible to the specified subfield of the journal club.
	Q8	Articles chosen were up to date.
	Q9	Competence of academic mentors and their conduct of meetings were sufficient.
	Q10	Meetings were understandable for me.
	Q11	Attendance to the journal club helped me improve my critical-thinking skills.
	Q12	My understanding and evaluation of methodology in research studies improved.
	Q13	Now, it is easier for me to determine the weaknesses and strengths of articles.
	Q14	My enthusiasm for future journal clubs or presentation activities increased.
	Q15	My presentation skills improved.
	Q16	My desire to be involved in research projects increased.
	Q17	My ability to understand and evaluate medical articles increased.
	Q18	I found it helpful to be able to interact with students from other medical schools.
	Q19	I would be more anxious if the meetings were in person.
	Q20	Online meeting platforms that were used for meetings were easy to use.
	QR1^a^	Meetings were not productive because of technical issues (internet speed, internet connectivity).
	QR2^a^	It was hard to get used to meetings being online.
**Preference questions**		
	P1	I wish there were journal clubs for subfields other than neurology, neurosurgery, neuroradiology, psychiatry, and neuroradiology.
	P2	I would prefer shorter meetings.
	P3	I would prefer longer meetings
	P4	I would prefer meetings with more people.
	P5	I would prefer meetings with less people.
	P6	Journal clubs are more beneficial for clinical students rather than preclinical students.
	P7	I would prefer online journal club meetings when the medical education becomes in person again.
	P8	I would prefer meetings were chaired by faculty members.

^a^Questions that were reverse-scored.

## Results

### Participants

A total of 13 applicants were accepted for neurology, 8 for neuroradiology, 8 for neuroscience, 9 for neurosurgery, and 12 for psychiatry, and they attended the meetings on a regular basis. Five participants, out of a total of 45, participated in two clubs. For each journal club, a member of our society’s general assembly was assigned as the academic mentor.

### Execution

Synchronous online journal club meetings were held every 2 weeks on a weekday since March 15, 2021, using Google Meet, Microsoft Teams, or Zoom. Each journal club’s meetings were approximately 1 hour long on average. First, an educational meeting was held to demonstrate how to present an article to participants. Following the first educational meeting, three articles were presented by three participants, who were selected 2 weeks earlier by academic mentors, at each of the subsequent meetings. In addition to presenters, each participant was expected to read the selected three articles in 2 weeks. A 10-minute presentation aided by slideshows was followed by a 10-minute discussion for each article. Academic mentors did not present any articles, but instead guided the discussions following each presentation. In some journal clubs, after the 1-hour meeting, participants and academic mentors would stay on the online platform to chat about their personal lives, which we believe contributed significantly to the motivation of participants and success of our journal club. The last meeting took place on June 29, 2021.

### Results of the Questionnaire

#### All Participants

Cronbach α was used to evaluate the internal consistency of the second section of the questionnaire in terms of reliability, which reached .84 (target value>.70).

**Figure 1:**
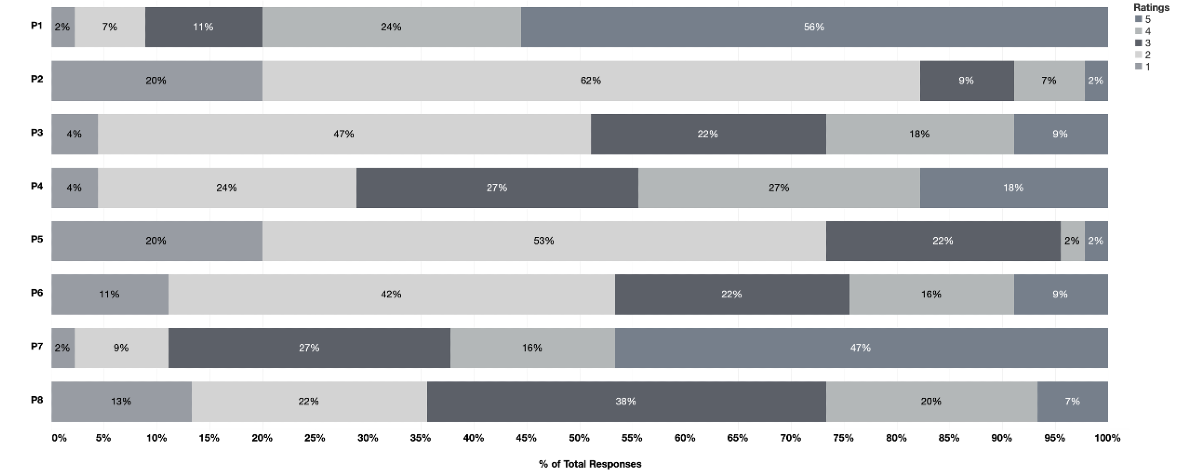
Responses to the preference-related questions (P1-P8; see Table 1 for complete descriptions of each item) on a 5-point Likert scale (1=completely disagree, 5=completely agree).

**Figure 2:**
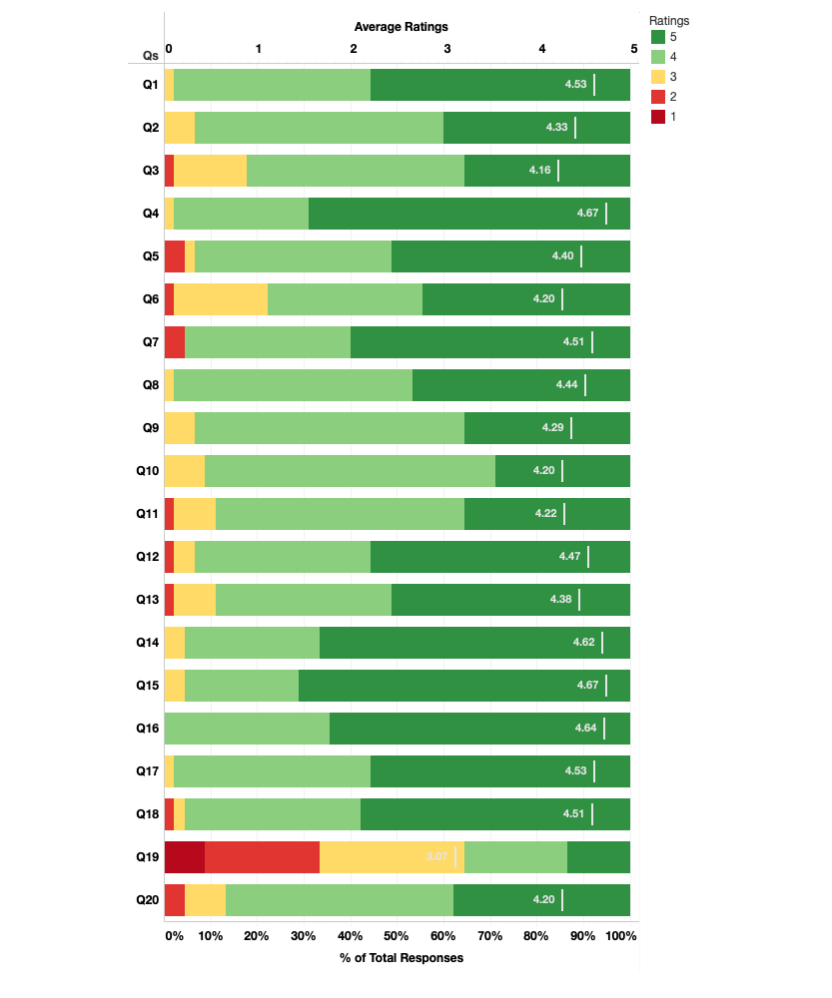
Average ratings for each question (see Table 1 for descriptions of each item) on a scale of 1 (strongly disagree) to 5 (strongly agree).

The journal club general contentment score was calculated by averaging all answers given by each participant to the second section of the questionnaire (Figure 1). The mean contentment score was 4.32, indicating that the journal club participants were very satisfied. The mean value for each item ranged from 3.07 to 4.67 (Figure 2).

Among the 45 participants, 27 (60%) were IUC students, whereas 18 (40%) were students from other universities, including Hacettepe University, Balikesir University, Hitit University, Kutahya Health Sciences University, Altinbas University, Istanbul University, Ankara University, Ataturk University, Gazi University, and Kahramanmaras Sutçu Imam University. In terms of the journal club general contentment score, there was no difference (*P*=.86) between IUC students (mean 4.32) and participants from other universities (mean 4.31).

Pearson correlations were performed to examine bivariate associations between the variables. Three items (reversed question one [QR1], reversed question 2 [QR2], and question 19 [Q19]) yielded a low Pearson correlation, indicating that these three items were scored significantly lower than other items by participants.

The first item with a low Pearson correlation was “Meetings were not productive because of technical issues (internet speed, internet connectivity)” with a mean score of 4 among the items with low Pearson correlation (reversed). This finding indicates that most participants were satisfied with their online connection; however, some participants encountered technical difficulties. The second item, with a mean score of 4.02 (reversed), was “It was hard to get used to meetings being online,” indicating that a few participants struggled to adapt to the online nature of the club. The last item was “I would be more anxious if the meetings were in person” with a mean score of 3.07. This finding implies that participants are unsure whether they would be more nervous in a face-to-face meeting as opposed to an online meeting.

#### Preclinical and Clinical Medical Students

In Turkey, medical education consists of 3 years of basic medical sciences courses (for preclinical medical students), 2 years of clerkships, and 1 year of internship (for clinical medical students). Participants included 38 preclinical medical students with 6 in their first year, 12 in their second year, and 20 in their third year. By contrast, there were only 7 clinical students, including 6 in their fourth year and 1 in their fifth year.

The independent-sample *t* test was used to compare the responses of these two groups, including responses to the preference questions. Significant differences between the answers of these two groups were found for the scores on only two items: preference 5 (P5) and preference 6 (P6). The first item that significantly differed between preclinical (mean 2) and clinical (mean 2.86) medical students (*P*=.01) was “I would prefer meetings with less people.” Despite the fact that neither preclinical nor clinical students prefer meetings in smaller groups, this finding suggests that clinical medical students are more likely to prefer small-group journal clubs. The second significantly different item between preclinical and clinical medical students was “Journal clubs are more beneficial for clinical students rather than preclinical students” (mean 2.50 and 3.71, respectively; *P*=.008). This finding suggests that clinical medical students think that participation in a journal club would be better after completing preclinical studies. However, we are very pleased with the positive attitude of preclinical participants, which indicates that they think the journal club has greatly benefited them even though they are still in the early stages of their medical education. As a result, we believe that preclinical medical students should be encouraged to participate in journal clubs.

The independent-samples *t* test was also used to compare the answers of IUC students and non-IUC students. There was no statistically significant difference in responses between IUC and non-IUC students (*P* values ranged from .08 to .94 for each item).

## Discussion

### Principal Results

This innovation widened students’ exposure to journal clubs, as many participants did not have this available at their university. Participation in our journal clubs is a valuable experience for our students, since involvement in a journal club was shown to enhance academic reading habits [10]. Participants’ responses indicated satisfaction with the way student academic mentors handled their responsibilities. The independent-samples *t* test revealed that the overall experience for IUC and non-IUC students was the same, indicating that institutions should be more welcoming of students from other institutions. Overall contentment scores suggested that peer-to-peer journal clubs could be satisfactory to participants and should be encouraged when the senior doctors or professors are unavailable to supervise.

### Strengths and Limitations

The COVID-19 pandemic has disrupted education, which, when combined with physical distance, has resulted in challenges in disseminating valid education for medical students, as well as social isolation. We present a student-led online synchronous journal club format that promotes education of medical students while also fostering peer interaction. During a time of societal stress, our journal club is unique in that it is entirely student-run, with participants consisting entirely of medical students, and it brings together a large number of medical students from all over Turkey, creating a venue for medical students, who are one of the most affected groups by self-quarantine [11].

Our survey provided valuable information on the essence of an online synchronous journal club for medical students. To obtain these valuable data, filling out the survey was required to obtain an online journal club certificate, which resulted in a 100% response rate. Some authors in the literature mentioned low response rates, which we believe could be resolved by making survey responses mandatory to receive a certificate [3,5,12].

Interstudent interaction across multiple institutions was generally accomplished through face-to-face conferences and courses, which were all completely cancelled during the pandemic. Our journal club had 45 participants from 11 universities, demonstrating that online events can partially compensate for the lack of in-person events for interinstitutional peer interaction. We believe that interinstitutional peer interaction is important not only for medical education but also for motivation and psychological well-being during this time of crisis. Furthermore, because we were aware that an online journal club was not available at other Turkey-based universities, we accepted non-IUC students to support these students, who did not have the same opportunity as IUC students. The mean score for the item “I would like to see journal clubs in other medical schools” among non-IUC participants was 4.56, indicating that such organizations are needed in other universities that participants attend. The multi-institutional nature of our journal clubs demonstrates that our online journal clubs were truly nationwide, with students from all over Turkey participating.

We believe that the small number of participants significantly contributed to the success of our journal club, because it is more difficult for participants to focus in large groups and for us to see how many participants are actively listening [3]. Furthermore, to achieve better results during meetings, participants in our journal club would turn on their webcams even if they were not presenting an article, making them more active and focused, especially during the discussion component of the meeting.

Since our journal club was entirely run by students, academic mentors in each journal club were members of the society’s board. Even though running a journal club was a huge responsibility for a pregraduate medical student, we believe that our mentors did a great job since overall participant contentment was very high. Another finding that supports our academic mentors’ success is that the item “I would prefer meetings be chaired by faculty members” received a mean score of 2.84. This finding suggests that our participants benefited sufficiently from their journal club experience while being supervised by student academic mentors. Furthermore, we believe that having this responsibility as a pregraduate medical student markedly improves leadership abilities. In addition, to handle this level of responsibility, our academic mentors had to thoroughly read the literature and internalize the particular topic, which significantly contributed to their medical and academic knowledge.

Learning necessitates an understanding of the subject matter, a willingness to put forth effort in studying, and the ability to control one’s education [9]. According to SDT, humans naturally tend to develop self-directed and autonomous behavior regulation [13]. Because motivation is the primary energy that drives learning, SDT is applicable at all levels of education, including our journal clubs [14]. The findings show that intrinsic motivation is linked to student achievement and well-being [15]. The students in our journal clubs already had an extrinsic motivation, obtaining a certificate, which is an essential aspect of learning, describing the psychological state apparent when individuals are driven to acquire outcomes apart from the pleasure innate in the behavior itself [15,16]. In addition to this extrinsic motivation, we hoped to increase our participants’ intrinsic motivation by giving them autonomy, which we believe positively impacted their successful presentations and general journal club experience.

Our study is not without limitations. First, the number of participants is insufficient to draw general conclusions. Perhaps beginning advertising earlier and more broadly could be beneficial in increasing the number of participants. However, as aforementioned, we do not intend to increase the number of participants in a particular journal club since participants were comfortable with the existing number. Instead, to accommodate more participants, the number of journal clubs should be increased. Second, since we do not have participant data to compare before and after implementation of the journal club, the survey only questioned participants’ perspectives rather than providing objective results. To overcome this obstacle in the future, participants should be evaluated in terms of academic knowledge such as literature searching and database usage, as well as the presentation of an academic paper before the first meeting and after the last meeting.

### Future Directions

As a society, we intend to continue our journal club for years to come. Even though medical education in Turkey is scheduled to be held face-to-face for the upcoming academic year, we intend to organize our journal club virtually for the upcoming academic year to minimize the COVID-19 risk and maintain the multi-institutional nature of our journal club. For this reason, to assess participants’ attitudes toward “online” journal clubs for the next year, one of the Likert-scale items was “I would prefer online journal club meetings when the medical education becomes in person again,” which resulted in a mean score of 3.96, indicating that the majority of our participants were pleased with the online format.

Given the short time between the announcement and the application deadline, there were more applicants than we expected. We intend to begin advertising for the upcoming academic year much earlier and increase the number of journal clubs, allowing us to contribute to the education of many more medical students whose institutions do not offer journal clubs.

Asynchronous Twitter journal clubs, in which participants contribute via tweets over a set period, have existed for some time, creating a diverse global forum for discussion [17,18]. We plan to implement an asynchronous Twitter journal club while keeping the synchronous format, allowing us to run a global journal club while also effectively advertising our journal club. If our asynchronous format can be successful, perhaps our synchronous format will become international, allowing medical students from all over the world to participate, particularly those whose medical schools do not provide such opportunities.

### Conclusion

In a time of crisis that isolated medical students from hospitals and universities, our journal club facilitated continued interaction between medical students by providing a platform for academic discussions. During the pandemic, students from all over Turkey regularly attended our club, which reduced social isolation and increased cross-institutional interaction. Our survey revealed that our participants were pleased with our journal club, which makes us very proud given that the club is entirely student-run. This experience, we believe, demonstrated that medical students can run a journal club on their own, and we hope that this paper serves as a guide for other organizations as they plan their journal clubs.

## Abbreviations

IUC: Istanbul University-Cerrahpasa
SDT: self-determination theory

## References

[1] Longhurst GJ, Stone DM, Dulohery K, Scully D, Campbell T, Smith CF (2020). Strength, Weakness, Opportunity, Threat (SWOT) analysis of the adaptations to anatomical education in the United Kingdom and Republic of Ireland in response to the Covid-19 pandemic. Anat Sci Educ.

[2] Linzer M (1987). The journal club and medical education: over one hundred years of unrecorded history. Postgrad Med J.

[3] Friedman KA, Herman SW, Fornari A (2019). Medical education using minimal technology: achieving professional development. Med Educ Online.

[4] Wood BP (1999). What's the evidence?. Radiology.

[5] Zavell AE, Greenberg JN, Alam M, Armbrecht ES, Maher IA (2017). A 30-minute, monthly, live, webinar-based journal club activity alters the self-reported behaviors of dermatologic surgeons. Dermatol Surg.

[6] Chetlen AL, Dell CM, Solberg AO, Otero HJ, Burton KR, Heller MT, Lakomkin N, Desouches SL, Smith SE (2017). Another time, another space: the evolution of the virtual journal club. Acad Radiol.

[7] Keet KA, Baatjes KJ, Venter RG, Wessels Q, Correia JC (2021). Development of a virtual journal club in anatomy: a responsive pandemic pedagogy. Med Sci Educ.

[8] Mark I, Sonbol M, Abbasian C (2021). Running a journal club in 2020: reflections and challenges. BJPsych Bull.

[9] Ten Cate TJ, Kusurkar RA, Williams GC (2011). How self-determination theory can assist our understanding of the teaching and learning processes in medical education. AMEE guide No. 59. Med Teach.

[10] Linzer M, Brown JT, Frazier LM, DeLong ER, Siegel WC (1988). Impact of a medical journal club on house-staff reading habits, knowledge, and critical appraisal skills. A randomized control trial. JAMA.

[11] Tahara M, Mashizume Y, Takahashi K (2021). Mental health crisis and stress coping among healthcare college students momentarily displaced from their campus community because of COVID-19 restrictions in Japan. Int J Environ Res Public Health.

[12] Musits AN, Mannix AL (2020). Synchronous online journal club to connect subspecialty trainees across geographic barriers. West J Emerg Med.

[13] Hoffman BD (2015). Using self-determination theory to improve residency training: learning to make omelets without breaking eggs. Acad Med.

[14] Biondi EA, Varade WS, Garfunkel LC, Lynn JF, Craig MS, Cellini MM, Shone LP, Harris JP, Baldwin CD (2015). Discordance between resident and faculty perceptions of resident autonomy: can self-determination theory help interpret differences and guide strategies for bridging the divide?. Acad Med.

[15] Howard JL, Bureau J, Guay F, Chong JXY, Ryan RM (2021). Student motivation and associated outcomes: a meta-analysis from self-determination theory. Perspect Psychol Sci.

[16] Ryan RM, Connell JP (1989). Perceived locus of causality and internalization: examining reasons for acting in two domains. J Pers Soc Psychol.

[17] Mehta N, Flickinger T (2014). The times they are a-changin': academia, social media and the JGIM Twitter Journal Club. J Gen Intern Med.

[18] Thangasamy IA, Leveridge M, Davies BJ, Finelli A, Stork B, Woo HH (2014). International Urology Journal Club via Twitter: 12-month experience. Eur Urol.

